# Malunion after midshaft clavicle fractures in adults: The current view on clavicular malunion in the literature

**DOI:** 10.3109/17453674.2010.480939

**Published:** 2010-05-21

**Authors:** Robert J Hillen, Bart J Burger, Rudolf G Pöll, Arthur de Gast, C Michael Robinson

**Affiliations:** ^1^Department of Orthopaedic Surgery, Academic Medical Centre University of Amsterdam; ^2^Department of Orthopaedic Surgery, Medical Centre Alkmaar; ^3^Department of Orthopaedic Surgery, VU University Amsterdam and Slotervaart Hospital Amsterdam; ^4^Department of Orthopaedic Surgery, Diakonessenhuis Utrechtthe Netherlands; ^5^Department of Orthopaedic Trauma, Royal Infirmary, EdinburghUK

## Abstract

This is an overview of the current literature on malunion after midshaft clavicle fracture. Anatomy, trauma mechanism, classification, incidence, symptoms, prevention, and treatment options are all discussed. The conclusion is that clavicle malunion is a distinct clinical entity that can be treated successfully.

## Introduction

The shoulder is a closed chain mechanism and constitutes the combined function of 4 joints: the sternoclavicular, the acromioclavicular, the scapulothoracic, and the glenohumeral joint. The function of each individual joint differs from the other 3 but the function of the 4 together is so intimately related that it is impossible to treat one of the constituents of the shoulder joint without influencing the mechanism of the others (Inman and Saunders 1946). 2 of the 4 joints are articulations of the clavicle; therefore, clavicle malunion affects the whole shoulder girdle. Symptomatic malunion after midshaft clavicular fractures has been recognized in the last 15 years to be a cause of shoulder dysfunction. Several authors have published reports about this condition (Eskola et al. 1986, Hill et al. 1997, McKee et al. 2003, Nowak et al. 2004, Ledger et al. 2005, Zlowodzki et al. 2005, Lazarides and Zafiropoulos 2006). Because reports about treatment of clavicular malunion with restoration of the length of the clavicle show good results (Simpson and Jupiter 1996, Bosch et al. 1998, Basamania 1999, Chan et al. 1999, McKee et al. 2003, 2004, Hillen and Eygendaal 2007, Rosenberg et al. 2007), this condition should be considered to be a distinct clinical entity. In this review, we make an analysis based on the current literature on clavicle malunion. Because of the limited amount of specific publications available on the subject and the low level of evidence, it is not a systematic review but rather a current concepts study. We cover the epidemiology of malunited midshaft clavicle fractures, as well as when to consider prevention of malunion of an acute midshaft clavicular fracture and when to treat a symptomatic malunion after closed treatment of a fracture of the clavicle midshaft. We also summarize the treatment options and possible complications.

### Anatomy

The clavicle is an S-shaped long bone with a cephalad caudad curvature (Andermahr et al. 2007, Huang et al. 2007). Attached to the medial side is part of the sternocleidomastoid muscle. On the lateral side, part of the deltoid and pectoralis major muscles are attached. The midshaft part of the clavicle is a transition zone between the flattened shape of the lateral part and the more tubular-to-triangular medial shape. It is the thinnest segment of the clavicle and is not stabilized by ligaments. Unlike the midshaft, both the lateral side and the medial side of the clavicle are stabilized by strong ligamentous and muscular structures. The midshaft is left relatively unprotected; thus, most fractures occur in the midshaft (Moseley 1968, Rowe 1968).

### Trauma mechanism

A fall onto or a direct blow to the shoulder, giving an axial compressive force on the clavicle, is the most common mechanism of injury for any clavicle fracture (Stanley et al. 1988, Nowak et al. 2000). Other mechanisms have been described but are rare, often as part of a more severe injury such as a floating shoulder (van Noort and van der Werken 2006). The midshaft or Edinburgh type 2B fractures tend to shorten when displaced. The displacement, and in turn shortening, is caused by unopposed muscular forces that occur when the shaft of the clavicle is fractured. Displacement of midshaft clavicular fractures is caused by the combined working of the sternocleidomastoid muscle pulling the medial fragment superiorly and posteriorly, and the pectoralis major muscle, the deltoid muscle, and gravity pulling the lateral fragment inferiorly and anteriorly. The net effect is a displacement of the ends of the fracture, with the lateral fragment lower than the medial fragment. The actual shortening is in turn caused by the medializing force components of the pectoralis, the trapezoid, and the latissimus dorsi muscles pulling the shoulder girdle medially. In our view, the shortening is therefore an ongoing process after a displaced fracture, although there is evidence that the amount of shortening between the first presentation of the fracture and (mal)union does not change substantially (Smekal et al. 2009). Other authors have seen a difference between initial shortening and the amount after (mal)union (Hill et al. 1997).

### Classification

**Figure 1.**
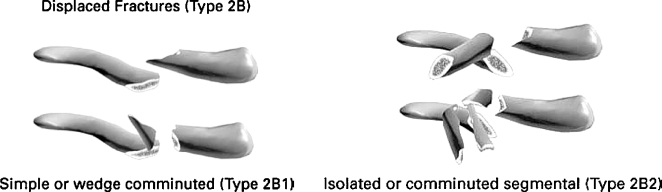
The displaced midshaft fractures in the Edinburgh classification for clavicle fractures.

Several classification systems have been suggested for clavicular fractures. The Edinburgh classification as suggested by Robinson (Robinson 1998, Khan et al. 2009) (Figure 1) is gaining popularity in the literature, and deals with the whole clavicle but is specific enough to deal with the individual problems for each segment. This paper will deal with the displaced midshaft or type 2B fractures. The Edinburgh classification system is the most valuable in terms of choosing therapy, as well as being of prognostic value for midshaft clavicular fractures. In the Allman classification, the clavicle is divided into 3 sections and numbered according to fracture incidence (midshaft I, lateral II, and medial III) (Allman 1967). This classification gives little information regarding choice of treatment or expectations about outcome. In this article, we only discuss the Allman type 1 fractures. The Orthopaedic Trauma Association (1996) suggested a classification in which the amount of fragments determined the classification of a midshaft fracture, varying from type A for simple fractures to type C for comminuted fractures. This classification system is not widely used. The classification systems as suggested by Neer (1963) and Craig (1990) are amendments on the Allman classification and are not of any interest for midshaft fractures.

### Epidemiology of midshaft clavicle fractures and incidence of malunion after such fractures

Clavicle fractures are common, with an incidence of up to 5% of all fractures in adults (Nordqvist and Petersson 1994, Robinson 1998, Nowak et al. 2000, Postacchini et al. 2002). Between 69% and 82% of these are midshaft fractures (Robinson 1998, Khan et al. 2009). Displacement occurs in about 73% of all midshaft clavicle fractures (Robinson 1998, Khan et al. 2009) and the frequency of nonunions is about 5%, but can be much higher in the group with displaced fractures (Robinson 1998, Robinson et al. 2004, Khan et al. 2009). Thus, of all midshaft clavicle fractures, about two-thirds will end up having some degree of malunion. Average shortening after a displaced fracture is about 1.2 cm, with a range of up to 3 cm (Eskola et al. 1986, Hill et al. 1997, Nordqvist et al. 1997). Shortening of more than 1.4–2 cm has been reported to be a critical deficit for development of a symptomatic malunion (Eskola et al. 1986, Hill et al. 1997, Nowak et al. 2004, 2005, Lazarides and Zafiropoulos 2006, Postacchini et al. 2009).

### Prevention of malunion after midshaft fracture in the acute phase

#### Nonoperative treatment.

Numerous closed treatment options have been described to immobilize and possibly re-align the dislocated fracture, and help in maintaining the alignment. However, almost all authors—to as far back as Hippocrates—have stated that maintaining the alignment after closed reduction of a displaced midshaft clavicle fracture is wishful thinking (Lester 1929, Adams 1939, Andersen et al. 1987, Khan et al. 2009). Methods still in current use are a simple sling or a figure-of-eight bandage, where the latter has been reported to be less comfortable and to offer no advantage over the simple sling (Andersen et al. 1987, Zlowodzki et al. 2005). Thus, closed treatment of a simple midshaft clavicle fracture should be with a simple sling (Eskola et al. 1986, Andersen et al. 1987, Nordqvist et al. 1998, Khan et al. 2009), but there is none operative measure to prevent a malunion after a displaced midshaft clavicle fracture.

#### Operative treatment.

The only way to prevent a malunion in a dislocated midshaft clavicle fracture is an open reduction with internal fixation or a percutaneous procedure. We will discuss the 2 types of fixation that are most commonly used: plate fixation and intramedullary fixation.

Plate osteosyntheses has frequently been reported to be a successful procedure for acute midshaft clavicular fractures (Rowe 1968, Zenni et al. 1981, Poigenfurst et al. 1992, Mullaji and Jupiter 1994, Bostman et al. 1997, Shen et al. 1999, Nowak et al. 2004, 2005, Coupe et al. 2005, Collinge et al. 2006, Russo et al. 2007) and there is some evidence that primary open reduction and internal fixation by means of plate osteosyntheses may be superior to primary closed treatment (Canadian Orthopaedic Trauma Society 2007). Plate osteosyntheses has the advantage of restoring length and alignment anatomically, and mechanically it is the strongest implant. Disadvantages are that it is more invasive than intramedullary options. Complications seen with plate osteosyntheses are infection, implant failure, implant loosening, refracture after implant removal, less frequent scar-related problems, and nonunion (Bostman et al. 1997, Smekal et al. 2009, Khan et al. 2009). A recent report of a prospective randomized trial described an incidence of adverse events of 37%; however, the proportion of complications in the nonoperative group was 63% (Canadian Orthopaedic Trauma Society 2007). Hardware has to be removed in about one third of cases after fracture healing because of prominence (Zlowodzki et al. 2005). There is a risk of neurovascular damage with screw placement (Galley et al. 2009). Both of these risks might be reduced by anterior-inferior placement of the plate (Kloen et al. 2002, Collinge et al. 2006, Kloen et al. 2009) but the superior plate position offers a more secure fixation (Iannotti et al. 2002, Celestre et al. 2008, Robertson et al. 2009). The reported rate of infection in a large systematic review was 1% (Zlowodzki et al. 2005), but in some reports the figure has reached 7.8% (Bostman et al. 1997). Most of the implant-related problems have now been addressed with specifically designed clavicular plates with angular stability. Plate osteosyntheses still remains the gold standard for osteosyntheses of fresh clavicular fractures (Kim and McKee 2008) and it is the most frequently used technique.

Percutaneous intramedullary osteosynthesis is another option for primary osteosynthesis of midshaft clavicular fractures. This technique has been described using different implants varying from Kirchner wires to different sorts of pins including elastic titanium nails (Ngarmukos et al. 1998, Grassi et al. 2001, Chu et al. 2002, Jubel et al. 2003, Frigg et al. 2009, Smekal et al. 2009). The technique can be used antegrade or retrograde. An extra incision to facilitate fracture reduction and guidance of the pin through the fracture site is usually necessary. Because of the narrow medulla and the curvatures of the clavicle, the challenge is for the implant to be flexible and small enough to be able to pass through the narrow medullary canal—and also to be rigid enough to offer the stability needed for the clavicle. These kinds of implants can help maintain the alignment but they offer no rotational stability, and with comminuted fractures, shortening of the clavicle can still occur. Advantages are minimal soft tissue damage and a less invasive procedure with theoretically little risk of damaging neurovascular structures. Disadvantages are higher risk of nonunion (Clavicular Midshaft Fractures 2004) and complications such as failure of the implant, wound problems over the point of entry, temporary brachial plexus palsy, and even implant migration in the direction of—or into—the great vessels (Nordback and Markkula 1985, Lyons and Rockwood 1990, Ring and Holovacs 2005, Strauss et al. 2007, Frigg et al. 2009).

**Figure 2.**
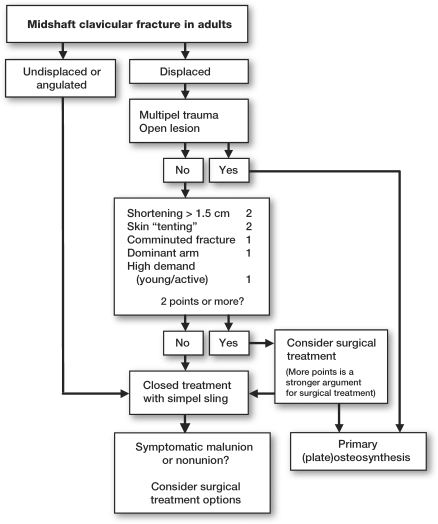
Suggested flow chart for making treatment decisions.

The cases in which primary osteosyntheses should be considered an optimal treatment are still under debate but displacement, shortening, comminution, and fractures on the dominant arm have all proven to be factors predisposing for an unfavorable outcome after conservatively-treated midshaft clavicular fractures (Eskola et al. 1986, Hill et al. 1997, Nowak et al. 2004, 2005, McKee et al. 2006). In the flow chart in Figure 2, we have listed factors for making a treatment decision based on the currently available evidence. By awarding points to each factor, we have tried to classify their relative importance in such a way that some factors alone are not enough to justify operative treatment while others can be.

### Symptoms and incidence of malunion after midshaft fracture

Early reports in the 1960s by Neer (1960) and Rowe (1968) formed the basis of the idea that few problems are to be expected after closed treatment of clavicular fractures regarding nonunion and functional problems. This was because the outcome was tested in terms of range of motion and radiographic fracture union. Rowe stated: “Fortunately for man, nature has endowed the clavicle with excellent reparative powers”. This applies to fracture union and unfortunately does not apply to the restoration of length and rotational deformities of the clavicle after a fracture. There have also been more recent studies that concur with the findings of Neer and Rowe (Nordqvist et al. 1997, Oroko et al. 1999). In the last decade, however, a number of studies using patient-based outcome scores have been published stating that malunion with shortening after a midshaft clavicle fracture may lead to symptoms such as pain, loss of strength, rapid fatigability, paraesthesiae of the arm and hand, and problems with sleeping on the back as well as cosmetic complaints (Hill et al. 1997, Ledger et al. 2005, Nowak et al. 2005, McKee et al. 2006, Rosenberg et al. 2007).

Complaints vary from mild to serious impairment in daily activities. Chan et al. (1999) reported atrophy of the trapezius muscle. Ledger et al. (2005) showed that there was loss of strength of the arm in patients with a shortening of the clavicle. He also noticed a reduced peak shoulder abduction velocity. Patients identified recreational activity as the area in which the functional loss was most evident. The reported incidence of unsatisfactory outcome after closed treatment of a displaced midshaft clavicular fracture has varied from 4.4% to 31% (Hill et al. 1997, Nowak et al. 2005, Lazarides and Zafiropoulos 2006), but the definition of unsatisfactory outcome has also varied between studies. Most authors have reported residual pain during activity or even at rest and loss of strength as main issues for an unsatisfactory outcome. Asking the opinion of the patient (for example: are you satisfied with the outcome?) has also been used as an outcome factor in several studies. Unpublished data from our own studies have shown that 30% of a consecutive series of patients with a dislocated fracture had a DASH score of above 20. The altered view on clavicular malunion has come about for several reasons. First, there have been better-designed studies, without inclusion of children, looking separately at specific problem groups within the Allman type 1 fractures (displaced fractures). Secondly, there is increased patient expectation regarding functional outcome after trauma. Lastly, but probably most importantly, outcome after malunion is now analyzed with a patient-based outcome score (Kim and McKee 2008, Smekal et al. 2009).

Many suggestions have been made as to what causes these symptoms:

#### Glenoid orientation/scapular winging.

Because of the shortened lever arm of the shoulder girdle, there is a change in orientation of the glenoid with winging of the scapula, which leads to functional problems of the shoulder in overhead movements. The change in orientation of the glenoid might also result in increased shear forces across the glenohumeral joint (Chan et al. 1999, Ledger et al. 2005, Andermahr et al. 2006). The increased protraction and tilt of the scapula can result in pain when lying on the back.

#### Muscular.

Shortening of the clavicle has a negative effect on muscle-tendon tension and muscle balance, which may result in loss of strength and endurability; this can be measured in patients with a short malunion (McKee et al. 2003, Ledger et al. 2005, McKee et al. 2006).

*Neurovascular problems/thoracic outlet syndrome* has been described after clavicular malunion, often associated with large callus formation. Patients complain of pain and rapid fatigue during overhead work (Chen and Liu 2000, Fujita et al. 2001, Onstenk et al. 2001, Connolly and Ganjianpour 2002).

#### AC/SC joint problems.

The change in resting angle of the SC joint after malunion (Ledger et al. 2005) results in a changed load of the AC and SC joint. Hill et al. (1997) reported AC arthrosis in patients after follow-up of malunited clavicular fractures.

It is likely that all of these explanations play a role. A decrease in length of the clavicle results in an alteration of the scapula position on the thoracic wall. Due to the ellipsoid shape of the thorax, changes in clavicular length result in nonlinear changes in scapula position: each additional millimeter of shortening results in an exponential increase in scapula malposition. This can lead to all of the above-mentioned problems.

### Treatment of symptomatic malunion after midshaft fracture

#### Nonoperative.

To our knowledge, no studies have been published on closed treatment of a malunited midshaft clavicle fracture, but it seems reasonable to start with nonoperative measures before considering surgical options. Closed treatment options can be physiotherapy (muscle strength, shoulder motion) or temporary pain medication. If a satisfactory result is not obtained, surgical treatment should be considered.

#### Operative.

Several reports on the operative treatment of malunited clavicular fractures have been published (Simpson and Jupiter 1996, Bosch et al. 1998, Basamania 1999, Chan et al. 1999, McKee et al. 2003, 2004, Hillen and Eygendaal, 2007, Rosenberg et al. 2007) (Table). Though all of them involved small series (the studies together reported on little over 40 patients) with a low level of evidence, all of them reported good results and satisfied patients. The ways of expressing the results differed, but the two largest series expressed the results in terms of DASH score. The mean reported decrease was between 20 (McKee et al. 2003) and 33 (Hillen and Eygendaal 2007). There was a large variance on the reported residual dysfunction. Most authors used a similar technique with or without a bone graft. It is useful to have a look at the original fracture X-ray to better understand the malunion. The technique described by Mc Kee et al. (2004) suggests an osteotomy through the original fracture plane. The patient is placed in a beach chair position under general anesthesia; the arm does not have to be draped free. The iliac crest is draped free when the need for bone grafting is expected. An oblique incision is made along the superior border of the clavicle. When the the skin and myofascial layers have been dissected, the malunion can be visualized. The original fracture plane is usually identifiable because of the typical pattern of the fracture ends relative to each other. The osteotomy is performed through this plane. If the original fracture cannot be easily recognized, an oblique sliding osteotomy can be performed. In both ends of the bone, the medullary canal is opened to hopefully restore blood supply to the osteotomy site. The length and alignment is restored with the opposite side as a reference for length measurement. If the “old fracture ends” can be recognized, these can also be used as a guide to restore length. Now the ends are fixated by means of either a pelvic reconstruction plate or a precontoured clavicle plate and compression is applied over the osteotomy. The plate is positioned most of the time on the postero-superior surface of the clavicle, especially precontoured plates (Huang et al. 2007), but the antero-inferior surface can also be used (Kloen et al. 2002, 2009, Collinge et al. 2006). The advantages of these plate locations are less prominence of hardware and reduced risk of neurovascular damage because the screws are directed away from vulnerable structures. An intramedullary device for stabilization has also been described (Basamania 1999, Chen and Liu 2000) After stable fixation, the shoulder can be mobilized immediately but forces should be limited to prevent hardware failure. Little is known about the timing of treatment, but correction osteotomy performed within 2 years of the fracture appears to give a better result than when performed a long time after fracture healing (Hillen and Eygendaal 2007). The risk of complications must be considered. Apart from hardware irritation requiring plate removal, due to infection, failure of fixation and nonunion are the most frequent complications reported, with frequencies of up to 20% (Table).

**Table:** Overview of results on corrective osteotomy after midshaft clavicle malunion

Study	No. of	Average follow-up patients	Type of scoring (months)	Average preoperative	Average postoperative	Plate removal	No. of complications
Bosch et al. 1998	4	24	Constant-Murley	?	C-M 89	?	0
Chan et al. 1999	4	24	Surgeons evaluation	?	?	3	0
Hillen and Eygendaal 2007	10	37	DASH	DASH 78	DASH 45	7	2
McKee et al. 2003	15	20	DASH	DASH 32	DASH 12	2	1
Rosenberg et al. 2007	2	41	Constant-Murley	?	C-M 56	?	?
Basamania 1999	9	>3	Surgeons evaluation	?	?	all (pin)	0

## Summary

The view on midshaft clavicle fractures has changed in the last 15 years. Adult patients with a displaced fracture have a higher number of nonunions than previously expected. Secondly, the outcome after union is now measured with patient-based outcome scores, which detect subtle loss of function in daily activities. Displaced fractures heal with some degree of shortening and they therefore result in malunion (or nonunion) unless treated operatively. Malunion can become symptomatic—with pain, loss of strength, rapid fatigue, numbness or parasthesiae of the arm and hand, and problems with sleeping on the back, as well as cosmetic complaints. Several mechanisms have been suggested to be responsible for these problems. Treatment can either be prevention in the acute phase, by means of primary osteosynthesis—or later when the symptomatic malunion is established, a correction osteotomy can be performed.
